# Phycospheric Bacteria Alleviate the Stress of Erythromycin on *Auxenochlorella pyrenoidosa* by Regulating Nitrogen Metabolism

**DOI:** 10.3390/plants14010121

**Published:** 2025-01-03

**Authors:** Jiping Li, Ying Wang, Yuan Fang, Xingsheng Lyu, Zixin Zhu, Chenyang Wu, Zijie Xu, Wei Li, Naisen Liu, Chenggong Du, Yan Wang

**Affiliations:** 1 Jiangsu Collaborative Innovation Center of Regional Modern Agriculture & Environmental Protection, Huaiyin Normal University, Huaian 223300, China; jipingli@hytc.edu.cn (J.L.); 1955240037@stu.hytc.edu.cn (Y.W.); 1955240006@stu.hytc.edu.cn (Y.F.); boomzip@163.com (N.L.); ducg@hytc.edu.cn (C.D.); 8202301082@hytc.edu.cn (Y.W.); 2 Jiangsu Key Laboratory for Eco-Agricultural Biotechnology Around Hongze Lake, Huaiyin Normal University, Huaian 223300, China; 3 School of Life Sciences, Huaiyin Normal University, Huaian 223300, China; 1902230008@stu.hytc.edu.cn (X.L.); 1906230090@stu.hytc.edu.cn (Z.Z.); 1902230010@stu.hytc.edu.cn (C.W.); 1902230035@stu.hytc.edu.cn (Z.X.); 4 College of Ecology and Environment, Nanjing Forestry University, Longpan Road 159, Nanjing 210037, China

**Keywords:** antibiotics, microalgae, nitrate reduction, microalgae–bacteria consortium

## Abstract

Macrolide pollution has attracted a great deal of attention because of its ecotoxic effects on microalgae, but the role of phycospheric bacteria under antibiotic stress remains unclear. This study explored the toxic effects of erythromycin (ERY) on the growth and nitrogen metabolism of *Auxenochlorella pyrenoidosa*; then, it analyzed and predicted the effects of the composition and ecological function of phycospheric bacteria on microalgae under ERY stress. We found that 0.1, 1.0, and 10 mg/L ERY inhibited the growth and chlorophyll of microalgae, but the microalgae gradually showed enhanced growth abilities over the course of 21 days. As the exposure time progressed, the nitrate reductase activities of the microalgae gradually increased, but remained significantly lower than that of the control group at 21 d. NO_3_^−^ concentrations in all treatment groups decreased gradually and were consistent with microalgae growth. NO_2_^−^ concentrations in the three treatment groups were lower than those in the control group during ERY exposure over 21 d. ERY changed the community composition and diversity of phycospheric bacteria. The relative abundance of bacteria, such as *unclassified-f-Rhizobiaceae*, *Mesorhizobium*, *Sphingopyxis*, *Aquimonas*, and *Blastomonas*, varied to different degrees. Metabolic functions, such ABC transporters, the microbial metabolism in diverse environments, and the biosynthesis of amino acids, were significantly upregulated in the treatments of higher concentrations (1.0 and 10 mg/L). Higher concentrations of ERY significantly inhibited nitrate denitrification, nitrous oxide denitrification, nitrite denitrification, and nitrite and nitrate respiration. The findings of this study suggest that phycospheric bacteria alleviate antibiotic stress and restore the growth of microalgae by regulating nitrogen metabolism in the exposure system.

## 1. Introduction

Macrolides can effectively inhibit bacterial protein biosynthesis by blocking peptidyl transferase activity in the 50s subunit of ribosomes; thus, this class of antibiotics is widely used to treat human and animal diseases [1]. The global consumption of antibiotics continues to rise every year, especially in low- and middle-income countries [2,3]. China consumes more than 160,000 tons of antibiotics annually, of which macrolides account for the highest proportion [4]. Some unmetabolized and unused macrolides eventually enter the aquatic environment through industrial wastewater and domestic sewage, resulting in high detection frequencies and concentrations in rivers, lakes, and sea water [5,6].

Erythromycin (ERY), as a typical macrolide, is commonly used to treat respiratory diseases in humans and animals, and it is reported to fall within the concentration range of ng/L–μg/L in surface water. For instance, the highest and mean concentrations of ERY in the Yangtze River (China) were recorded to be 808 and 296 ng/L [7]; meanwhile, ERY concentrations ranging from 10 to 350 ng/L were reported in European rivers [8]. Additionally, recent studies have demonstrated that ERY exhibits ecotoxicity to aquatic organisms, such as fish, microalgae, and duckweed [9,10,11]. Microalgae have attracted the most concern among these because they are more sensitive to environmental stress, especially antibiotic exposure. Concentrations of 50–90 μg/L ERY were found to inhibit the growth of *Raphidocelis subcapitata* [12]; our previous study also investigated the growth effect of ERY on *Auxenochlorella pyrenoidosa* (H. Chick) Molinari and Calvo-Pérez (*A. pyrenoidosa*). The 96 h median effect concentration was 7.37 mg/L [10]. Many studies have demonstrated that ERY exposure induces an imbalance in reactive oxygen species, leading to an excess of reactive oxygen species that subsequently cause lipid peroxidation in both the cell membrane and the organelle membrane; finally, the metabolic functions related to the growth of microalgae cells have been found to be affected by ERY stress [12,13,14]. Transcriptomic analysis has revealed that ERY regulates the expression of genes involved in porphyrin and chlorophyll metabolisms, DNA replication, energy metabolism, nucleoside metabolism, and ATP-binding cassette transporters [15,16,17]. Meanwhile, previous studies have indicated that defense mechanisms, such as the antioxidation system, the photosystem, and gene regulation, enhance the resistance of microalgae and, to an extent, alleviate the damage caused by antibiotic stress [18,19]. However, few studies focus on the effects of extracellular factors on the antibiotic resistance of microalgae. Our previous study detected symbiotic bacteria in microalgae in non-strictly sterile toxicity tests under ERY exposure. The microalgae–bacteria consortium has also been demonstrated to be more resistant to environmental stress [20,21]. The phycosphere is widely considered to be a microenvironment where microalgae interact with bacteria, and it is filled with metabolites from both, such as nutrients and signaling molecules [22,23]. Therefore, we suspect that phycospheric bacteria may influence the response of microalgae to ERY stress.

Microalgae and phycospheric bacteria surrounding microalgae form a species- and environment-specific symbiosis system; they provide each other with nutrients and essential growth factors [24,25]. Many studies have indicated that the microalgae–bacteria consortium increases the adaptability of microalgae to environmental stress. For example, the presence of bacteria enhanced the resistance of two microalgae, *Thalassiosira delicatula* and *Isochrysis galbana*, to stresses caused by metals and pesticides; the bacteria induced the microalgae to synthesize more metabolites to defend against environmental stress rather than growth [26]. Under the stress of antibiotics, the microalgae–bacteria consortium produces more extracellular polymeric substances to enhance its ability to adsorb antibiotics; then, antibiotics are biodegraded more efficiently [20,27]. In addition, phycospheric bacteria were reported to enhance the nutrient exchange, intercellular communication, and metabolites’ stimulation of the microalgae–bacteria consortium, leading to the development of bioactivity, tolerance, and metabolism in symbiosis systems [28,29,30]. In the presence of phycospheric bacteria, microalgae exposed to tetracycline and norfloxacin suffered lower rates of lipid peroxidation and cell structural damage; moreover, the microalgae–bacteria consortium showed a stronger efficiency of nitrogen metabolism and antibiotics removal compared to when it was exposed to antibiotics alone [31]. Because of the specific ecological relationship between bacteria and microalgae, phycospheric bacteria directly and indirectly modulate microalgae nutrition and adaptability [32]. For example, microalgae cannot utilize proline as a nitrogen source, but *Methylobacterium* can metabolize L-proline into ammonium to feed *Chlamydomonas*; together, they establish a stable symbiotic system [33,34]. The elimination efficiency of nitrogen by phycospheric bacteria under antibiotic stress is obviously affected by their species composition [35]; dynamic changes in the microbial community structure regulate the adaptation of the microalgae–bacteria consortium to pollutant stress [24]. Therefore, phycospheric bacteria may enhance the resistance of microalgae to antibiotics and facilitate the nitrogen metabolism of microalgae. This idea requires further verification in order to accurately assess the ecotoxicity of antibiotics to microalgae in aquatic environments.

As noted in many studies, *A. pyrenoidosa* is a common primary producer in aquatic environments and is commonly used in toxicity tests for antibiotic exposure due to its high sensitivity and susceptibility to antibiotics [36,37,38]. Previous toxicity tests have normally required the use of a breathable sealing film to isolate the inside of the conical flask from external airborne environmental pollutants (bacteria and particulate matter), but our pre-experimental studies found that in an open exposure system without a sealing film, bacterial communities colonized the surface of microalgae cells, and their presence appeared to enhance the resistance of microalgae to antibiotics. Therefore, this study was performed based on our previous toxicity test of ERY to *A. pyrenoidosa* [39]. The research undertaken to verify the above conjecture proceeded as follows: (1) an investigation of the toxic effects of ERY on the growth and chlorophyll synthesis of microalgae; (2) an investigation of the nitrogen metabolism and related enzyme activity of microalgae; and (3) an exploration of the community composition and metabolic and ecological functions of phycospheric bacteria in the process of microalgae exposure to ERY. This study aims to provide a scientific foundation for determining the resistance mechanism of microalgae with the assistance of phycospheric bacteria.

## 2. Materials and Methods

### 2.1. Chemicals

ERY with a purity greater than 98% was purchased from Macklin (Shanghai, China). Analytical-grade chemicals used to prepare the BG11 culture medium, such as NaNO_3_, CaCl_2_·2H_2_O, and NaCO_3_, were all purchased from Macklin (Shanghai, China), and the preparation method is shown on the website of the Freshwater Algae Culture Collection at the Institute of Hydrobiology, Chinese Academy of Sciences (https://algae.ihb.ac.cn/ (accessed on 1 September 2022)). Ethanol and hydrochloric acid were purchased from the Sinopharm Group Chemical Reagent Co., Ltd., Shanghai, China. All of these chemicals were dissolved or diluted using ultrapure water (18.2 MΩ).

### 2.2. Experimental Procedure

Microalgae, *A. pyrenoidosa*, were purchased from the Freshwater Algae Culture Collection at the Institute of Hydrobiology (Chinese Academy of Sciences, Beijing, China). Then, the microalgae were precultured according to the instructions. The illumination incubator was set at 25 ± 1 °C and 3000 lux, with 12 h of darkness and 12 h of light within a 24 h period. The precultured microalgae strains were used for the batch experiment until the microalgae density reached 10^7^ cells/mL.

As shown in Table 1, the batch experiment for 21 d was performed in 250 mL sterile conical flasks. Successively added into the conical flasks were 50 mL of the BG11 culture medium of double concentration (BG11 × 2), several volumes of ERY stock solution (25 mg/L, sufficiently dissolved), several volumes of sterile ultrapure water, and 10 mL of the precultured microalgae strain. The initial exposure concentrations of ERY were set at four levels, 0, 0.1, 1.0, and 10 mg/L, these being the control group, and the low, moderate, and high concentration levels (approximately 96 h EC_50_). All treatments were conducted in triplicate, and the samples were subsequently incubated in illumination incubators for 21 d. The culture conditions were the same as those used for the preculture. All of the treatments were harvested for further determinations at 0, 3, 7, 10, 14, and 21 d.

### 2.3. Determination of Microalgae Growth and Chlorophyll Content

The growth of the microalgae was represented as the cell density at each sampling time. According to a previous study [40], the cell density was determined using microscopy combined with absorbance at 680 nm, and the results were calculated using Equation (1):

Cell density (10^6^ cells/mL) = 109.56 × A_680_ − 4.6501 (R^2^ = 0.9901) (1)

The total chlorophyll content was measured according to the previously described method, with appropriate improvements [41]. Briefly, the microalgae suspension (5 mL) was centrifuged at 8000 rpm, and then the supernatant was discarded. The collected microalgae particles were resuspended in 5 mL of 95% ethanol. The samples were held in the dark at 4 °C for 24 h and then centrifuged at 10,000 rpm for 10 min. The absorbance of the supernatant containing chlorophyll was determined at 649 and 665 nm. The total chlorophyll content (mg/L) was calculated using Equation (2):

Chlorophyll content = 20.04 × A_649_ + 6.10 × A_665_ (2)

### 2.4. Determination of NO_3_^−^ and NO_2_^−^

The concentration of NO_3_^−^ in the culture medium over the 21 d was measured using the ultraviolet absorption spectroscopy method [42]. Briefly, the microalgae suspension (5 mL) was centrifuged, and then the supernatant was collected after filtering using a 0.45 μm filter membrane. Hydrochloric acid and ammonium sulfamate were added to eliminate interference, and then the concentration of NO_3_^−^ was measured using a spectrophotometer at 220 and 275 nm.

The concentration of NO_2_^−^ in the culture medium over the 21 d was measured using a nitrite assay kit (Nanjing Jiancheng Bioengineering Institute, China). Briefly, the above-mentioned supernatant obtained via filtration was used for the determination of NO_2_^−^. The measurement was performed according to the operation instructions. The NO_2_^−^ formed a light-red azo compound with the chromogenic agent, and then the concentration of NO_2_^−^ was measured using a spectrophotometer at 550 nm.

### 2.5. Determination of Nitrate Reductase Activity

The activity of nitrate reductase in the microalgae cells was determined using a nitrate reductase assay kit purchased from the Nanjing Jiancheng Bioengineering Institute (Nanjing, China). Briefly, 30 mL of the microalgae suspension was centrifuged at 8000 rpm for 10 min, and then the microalgae particles were washed three times to remove the phycospheric bacteria [43]. The collected microalgae particles (without bacteria) were resuspended in the phosphate buffer (10 mM, pH = 7.3). Then, the resuspended microalgae were sonicated at 900 W for 10 min, with the working and intermittent time of the ultrasonic crusher set to 2 s. The obtained crude enzyme was used to determine the activity of nitrate reductase after it was centrifuged, and the operation procedure was performed according to the operation instructions.

### 2.6. Determination of the Communities and Ecological Functions of Phycospheric Bacteria

In the open antibiotic exposure system, bacterial communities from the environment quickly colonized the surface of microalgae cells, forming phycospheric bacteria. The determination of phycospheric bacterial communities was carried out by the Majorbio Biotechnology Company (Shanghai, China). In general, bacterial genomic DNA from the phycosphere of the microalgae was harvested at 3, 7, 10, 14, and 21 d, and DNA was extracted using the E.Z.N.A.^®^ soil DNA Kit (Omega Bio-tek, Norcross, GA, USA). Then, the extracted DNA was analyzed on 1% agarose gel, and its concentration and purity were assessed using a NanoDrop 2000 UV-vis spectrophotometer (Thermo Fisher Scientific, Waltham, MA, USA). The V3–V4 region of the bacterial 16S rRNA gene was amplified using an ABI GeneAmp^®^ 9700 PCR thermocycler (ABI, Foster City, CA, USA) with specific primer pairs: 338F (5′-ACTCCTACGGGAGGCAGCAG-3′) and 806R (5′-GGACTACHVGGGTWTCTAAT-3′). The PCR amplification reaction of each sample was performed in triplicate, and then the PCR products were extracted from the agarose gel (2%) and subsequently purified. The purified products were then mixed and sequenced using the paired-end method on an Illumina MiSeq PE300 platform (Illumina, San Diego, CA, USA). The raw data obtained from the high-throughput sequencing of the phycospheric bacteria were deposited into the NCBI Sequence Read Archive database, under accession number PRJNA1147877.

### 2.7. Statistical Analysis

The significance level between different treatments was determined using SPSS 18.0 software, and treatments were considered to have a statistically significant difference when the *p*-value was less than 0.05. The community composition and diversity were analyzed based on sequencing data using the Majorbio Cloud Platform (www.majorbio.com (accessed on 5 December 2022)). The metabolic and ecological functions of the phycospheric bacteria were analyzed using the PICRUSt2 and FAPROTAX databases (Functional Annotation of Procaryotic Taxa); this procedure was easily performed and graphed on the Majorbio Cloud Platform.

## 3. Results and Discussion

### 3.1. Effects of ERY on Microalgae Growth and Chlorophyll

**Figure 1:**
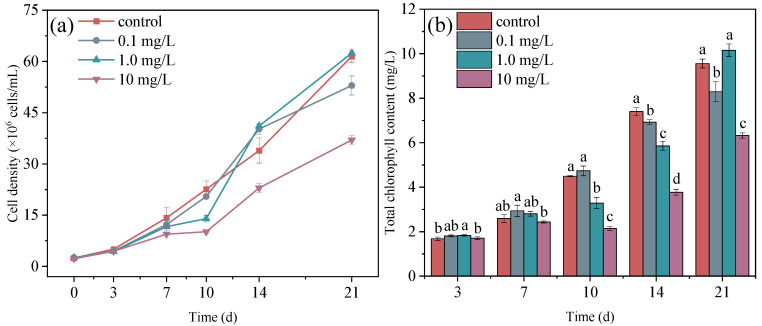
Cell density (**a**) and total chlorophyll content (**b**) of microalgae in the control group and the 0.1 mg/L, 1.0 mg/L, and 10 mg/L ERY exposure groups for 21 d. The error bar represents the standard deviation of three repetitions. Significant differences were judged according to *p* < 0.05 and are represented as different letters.

During the 21 days of microalgae exposure to ERY, microalgal growth was obviously affected by the ERY concentration, especially after the third day (Figure 1a). During the first 10 d, the cell density of the microalgae was inhibited. However, after 14 d of treatment with 0.1 mg/L ERY, there was an increase in growth, followed by a subsequent inhibition at 21 days. A similar trend was observed in the 1.0 mg/L treatment, with slight growth promotion compared to the control group after 10 d. The cell densities were 87.00%, 82.17%, 61.88%, 121.19%, and 101.52% of that of the control group at 3, 7, 10, 14, and 21 d, respectively. However, microalgal growth was consistently inhibited in the 10 mg/L treatment group during the 21 d of ERY exposure. The cell densities were 88.31%, 66.45%, 44.76%, 67.73%, and 60.17% for the control group, but the growth inhibition rate decreased in the later period. Many previous studies have indicated that ERY exposure induces the production of reactive oxygen species, which further results in the lipid peroxidation of the cell membrane and organelle membrane in microalgae. Meanwhile, the antioxidant system of microalgae protected against the oxidative damage caused by reactive oxygen species [39,44]. When antioxidant enzymes could not clear reactive oxygen species in a timely manner, microalgal growth was inhibited. Instead, microalgae growth was promoted [13,45]. In addition, the interaction between antioxidant systems and toxic stress is also related to exposure time. Long-term exposure may enhance the resistance of microalgae to antibiotics [46]. Antibiotics may also be converted into less toxic degradation products [18,39]. Microalgae gradually acquired resistance to antibiotics during long-term exposure in this study, and this explains the recovered growth ability of microalgae during the late exposure period.

The effects of erythromycin on the chlorophyll synthesis of microalgae in the early and late stages were also different (Figure 1b). At three and seven days of ERY exposure, the total chlorophyll contents in the treatments of 0.1 and 1.0 mg/L were higher than in the control group. The increase in ERY concentration had a significant effect on chlorophyll synthesis at 10 and 14 d: that is, the total chlorophyll content decreased as the ERY concentration increased. At 14 days, the total chlorophyll contents in the treatments of 0.1 mg/L, 1.0 mg/L, and 10 mg/L were 93.58%, 79.13%, and 51.00% of that of the control, respectively. Generally, the response of chlorophyll to ERY was consistent with microalgal growth. Compared to the control group, the total chlorophyll content was slightly higher in the 1.0 mg/L treatment group, but significantly lower in the 0.1 mg/L and 10 mg/L treatment groups. The initial energy required for microalgal proliferation comes from photosynthesis, which relies on chlorophyll, so microalgal growth ability is closely related to chlorophyll content [47]. Previous studies have indicated that ERY regulates the expression of genes related to chlorophyll biosynthesis and the photosynthesis process, such as *chlE*, *bchH*, and *lhca1*. These genes play important roles in photosystems Ⅰ and Ⅱ [16,48]. The changes in gene expression related to photosynthesis resulted in a change in chlorophyll content and ultimately affected microalgal growth abilities. These findings provide a reasonable explanation for the consistency of growth and chlorophyll content in this study.

### 3.2. Effects of ERY on the Nitrate Reductase Activity of Microalgae

**Figure 2:**
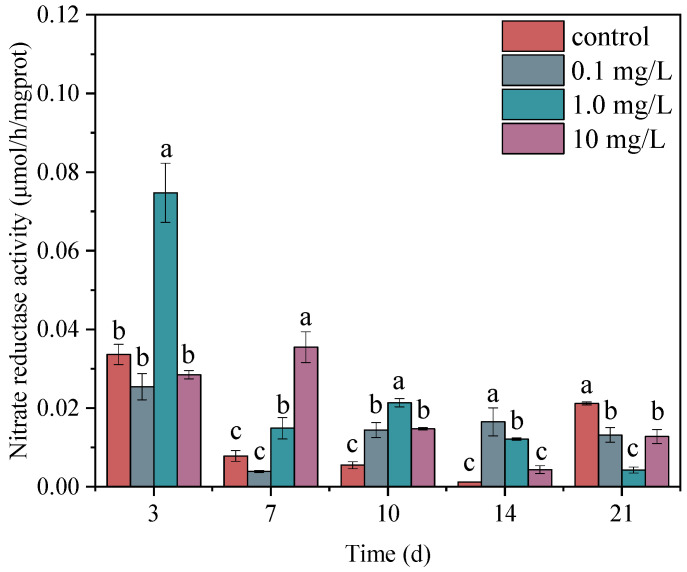
Nitrate reductase activity of microalgae under exposure to 0.1, 1.0, and 10 mg/L ERY. The error bar represents the standard deviation of three repetitions. Significant differences were judged according to *p* < 0.05 and are represented as different letters.

Antibiotic exposure also affects microalgal cell growth by inhibiting the activities of metabolic enzymes. Antioxidant enzymes, such as superoxide dismutase and catalase, were the most relevant indicators [49,50]. Enzymes related to carbon metabolism, carbonic anhydrase and ribulose bisphosphate carboxylase, were also observed to evaluate the effect of antibiotics on microalgae growth [51]. It is well known that nitrogen metabolism in microalgae is another an important way to affect cell growth [52,53]. Nitrate reductase in microalgae enzymatically reduces nitrate to nitrite; then, the nitrite is further converted into bioavailable ammonia nitrogen. This study observed the changes in nitrate reductase activity to investigate the effects of ERY on nitrogen metabolism in microalgae (Figure 2). At three days, the nitrate reductase activity of the microalgae in the 1.0 mg/L treatment group was significantly higher than that in the control group. As the exposure time progressed, the nitrate reductase activities in the treatment groups gradually increased, e.g., in the 1.0 and 10 mg/L treatments at 7 d, and in the three treatments at 10 d. However, the nitrate reductase activities in the treatment groups were significantly lower than those of the control group at 21 d. Many previous studies have reported that microalgae show a hormesis effect in a suitable range of antibiotic concentrations and exposure times. In other words, microalgae can enhance some metabolic functions within a certain concentration range to compensate for suppressed functions, and this concentration range is time-dependent [54,55,56]. The increase in nitrate reductase activities at different times may also be caused by the hormesis effect at this ERY concentration. Under the stress of ERY, similar to the self-defense mechanism of the antioxidant system, the nitrogen metabolism system of the microalgae also struggled to upregulate gene expression and biosynthesize intermediate products to ensure the growth of microalgae cells [57,58]. In this study, the increased nitrate reductase activities accelerated the conversion of nitrate to ammonium, and the available nitrogen contributed to microalgae growth. The model microalgae *Chlamydomonas* was also reported to exhibit the same mechanism of nitrogen conversion and utilization [59]. In addition, nitrate reductase has been suggested to promote the production of nitric oxide, a signal molecule that maintains the homeostasis of nitrogen metabolism in microalgae cells [60]. The increased nitrate reductase activities were consistent with the reduced growth inhibition or even growth promotion in the first 14 days. However, long-term exposure to antibiotics may lead to metabolic dysfunction, where growth-related chlorophyll synthesis and nitrogen metabolism are severely inhibited [61].

### 3.3. Changes to NO_3_^−^ and NO_2_^−^ in the Culture Medium

**Figure 3:**
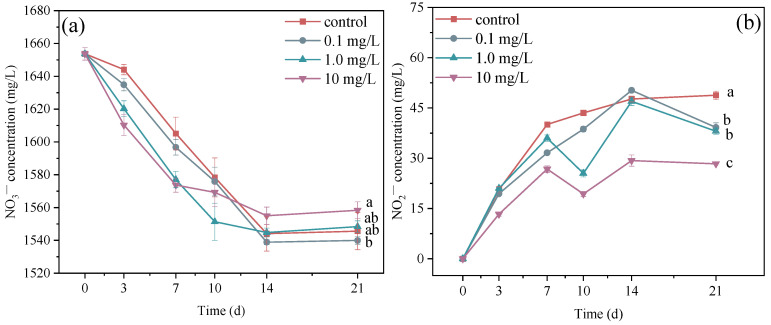
Changes in NO_3_^−^ (**a**) and NO_2_^−^ (**b**) concentrations in the culture medium under ERY exposure concentrations of 0.1, 1.0, and 10 mg/L ERY. Significant differences were assessed according to *p* < 0.05 and are represented as different letters.

The activity of nitrogen metabolism in microalgae cells is crucial for cell growth, and the occurrence state of nitrogen in the extracellular environment produced by the microalgae–bacteria consortium should not be ignored [62,63]. This investigation was conducted in an artificial medium. The initial occurrence state of nitrogen was NO_3_^−^ with a concentration of 1653.70 mg/L (Figure 3a). In the first 10 d of ERY exposure, the consumption rates of NO_3_^−^ in the three treatments were higher than those of the control group; then, the NO_3_^−^ concentration decreased slowly and remained at a relatively stable level. For example, the NO_3_^−^ concentrations in the 0.1 mg/L treatment were 1634.87, 1596.72, 1575.85, 1538.91, and 1539.99 mg/L at 3, 7, 10, 14, and 21 d, respectively. NO_3_^−^ concentrations decreased gradually with microalgae growth, but there were differences in the consumption rate and total consumption between these treatments. For example, the residual NO_3_^−^ concentration in the 10 mg/L treatment group was significantly higher than that in the other treatment groups. With the open antibiotic exposure, bacterial communities rapidly colonized the surface of microalgae cells. With the increase in microalgae density, the denitrification process instigated by the denitrifying bacteria was strengthened under dark conditions, and some of the NO_3_^−^ was converted into NO_2_^−^. NO_2_^−^ concentrations in these treatments also varied during the 21 d (Figure 3b). In the first 10 d of ERY exposure, the production rates of NO_2_^−^ in the three treatment groups were lower than that in the control group. After 10 d, the NO_2_^−^ concentration in the 0.1 treatment and control groups increased slowly in general, but there were several significant reductions in the three treatments—for instance, at 10 d for the 1.0 mg/L treatment and at 21 d for the 0.1 mg/L treatment. In general, NO_2_^−^ concentrations in the three treatment groups were significantly lower than that in the control group during ERY exposure across the 21 d. In the early stages of ERY exposure, microalgae assimilated nitrogen in excess from water; some of this nitrogen was used for cell growth, while the rest was stored in the cells in different forms for stress prevention [64,65]. Nitrate reductase is involved not only in nitrogen conversion but also in nitrogen storage in cells [66]. Increased environmental stress or interspecific competition may reduce the cells’ capacity to absorb nutrients from the water environment, and the stored nitrogen can then be used in microalgae growth. On the other hand, the decreased NO_2_^−^ concentration may be converted into nitrate under the action of nitrifying bacteria, contributing to the nitrate concentration [67]. In addition, nitrogen-fixing bacteria can also fix nitrogen from the air for the microalgae–bacteria consortium [68]. The three treatment groups consumed more NO_3_^−^ than the control group in the earlier stage, but the production of NO_2_^−^ was lower in the culture medium; this indicates that microalgae cells exposed to ERY absorbed and stored more NO_3_^−^ for amino acid biosynthesis [69]. This was consistent with the decreasing growth inhibition during the earlier stage. During the later stage, the consumption of NO_3_^−^ was positively correlated with microalgae growth; after all, NO_3_^−^ was the only source of nitrogen in the beginning. Microalgae growth was obviously affected by the antibiotic concentration; this viewpoint is supported in this study and in many previous studies [50,70]. In addition, microalgae growth has also been reported to be inhibited by the presence of NO_2_^−^ [71]. In this study, NO_2_^−^ concentration increased with exposure time, and microalgae growth was inhibited by both ERY and NO_2_^−^. In general, NO_2_^−^ concentrations were lower in the treatments with high ERY concentrations, which may be related to the abundance of bacteria with denitrification functions in the high-concentration treatments [72,73]. These findings indicate that the conversion rate of NO_3_^−^ to NO_2_^−^ was lower in the treatment with a high concentration of ERY, meaning that more NO_3_^−^ could be absorbed for microalgae growth.

### 3.4. Community Composition of Phycospheric Bacteria

**Figure 4:**
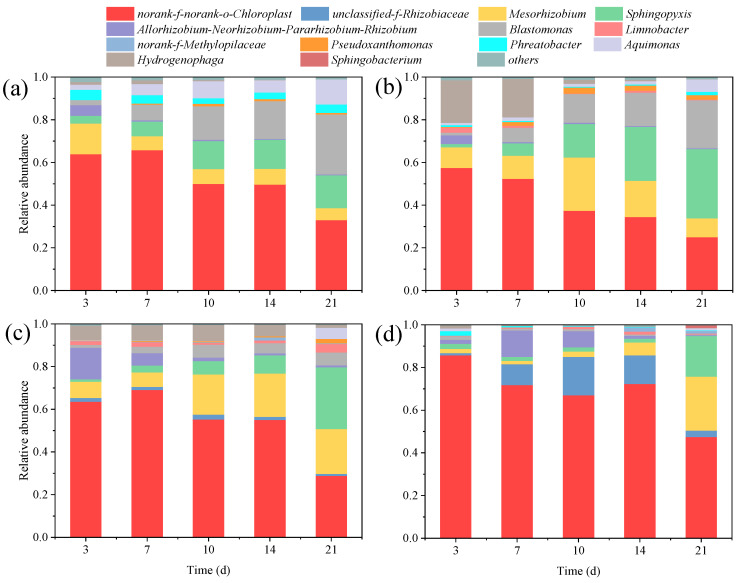
Community composition of phycospheric bacteria at the genus level in the control group (**a**), and in the 0.1 (**b**), 1.0 (**c**), and 10 (**d**) mg/L ERY treatment groups across 21 days. Only bacterial genera with an abundance greater than 1% are shown.

During the period of ERY exposure, samples of phycospheric bacteria were collected at 3, 7, 10, 14, and 21 d. The 16s rRNA gene of the phycospheric bacteria was amplified and sequenced to analyze the characteristics and function of the bacterial community. In total, 3,575,214 effective gene sequences were obtained from 60 samples, and the average length was 407. Accordingly, a total of 13 bacterial genera with an abundance greater than 1% were identified in the bacterial community around microalgae exposed to ERY (Figure 4). At the genus level, the compositions of phycospheric bacteria were very different between each treatment at 3, 7, 10, 14, and 21 d. *Norank-f-norank-o-Chloroplast* is a sequence related to chloroplasts and is often present in the composition of bacterial communities. The other 12 bacterial genera with a relative abundance greater than 1% showed obvious differences between the treatments; for instance, *unclassified-f-Rhizobiaceae* was not detected in the control and the 0.1 mg/L ERY treatment, but it was detected in the higher-concentration treatments (1.0 and 10 mg/L) with a relative abundance of 0.85–2.27% and 1.06–17.96%, respectively. The relative abundance of *Mesorhizobium* in the control group and the 0.1 mg/L treatment group decreased with time but increased in the higher-concentration treatment. *Sphingopyxis* and *Aquimonas* increased, but *Allorhizobium-Neorhizobium-Pararhizobium-Rhizobium* decreased across the 21 d in the three treatment groups. The relative abundance of *Blastomonas* increased from 2.45% to 28.06%, from 1.22% to 22.52%, and from 1.30% to 5.95 in the control group, the 0.1 treatment group, and the 1.0 mg/L treatment group, respectively. However, the relative abundance of *Blastomonas* decreased from 1.58% at 3 d to 0.25% at 21 d. The relative abundance of *Hydrogenophaga* also gradually decreased over the 21 d in all treatment groups. The proportion of other bacterial genera was small and showed no obvious changes.

In an exposure system of ERY, the community composition of phycospheric bacteria around microalgae may be affected by many factors, such as light, nutrients, interspecific competition, and environmental stress [74,75]. In this study, the detected number of phycospheric bacteria at the genus level was significantly lower than that of the environmental samples; this may be related to the antibacterial effect of antibiotics [76,77]. The abundance of bacteria containing sequences related to chloroplasts decreased gradually in all of the treatments, perhaps due to the growth of the dominant species, *A. pyrenoidosa*. The growth of microalgae enhanced not only nutrient competition but also competition with these bacteria for light [78]. With the increase in exposure time, the increase in *Mesorhizobium*, *Sphingopyxis*, *Blastomonas*, and *Aquimonas* may be benefit from microalgae growth. Higher microalgae density indicates stronger photosynthesis and a larger oxygen supply in the microalgal–bacterial symbiosis system [79]. In addition, extracellular organic matter produced by microalgae metabolism may also provide suitable nutrients for these bacteria. The community composition of phycospheric bacteria was closely related to the metabolites secreted by microalgae, such as phytosphingosinen and α-Linolenic acid [80,81]. Similarly, previous studies have found that indole acetic acid, a kind of auxin produced by *Chlamydomonas*, provided a nitrogen source for bacteria, allowing bacterial communities to thrive in a coculture system [82]. On the other hand, these phycospheric bacteria also took part in the nutrient transport and uptake of microalgae; they transformed nutrients into more available states, such as the nitrogen metabolism process induced by nitrifying and denitrifying bacteria [72], and nitrogen-fixing bacteria, such as *Rhizobiaceae* and *Mesorhizobium* [68]. These inevitably affect the growth of microalgae. Therefore, it is necessary to further investigate the diversity and ecological function of phycospheric bacteria under ERY exposure.

### 3.5. Diversity Analysis of Phycospheric Bacteria

**Figure 5:**
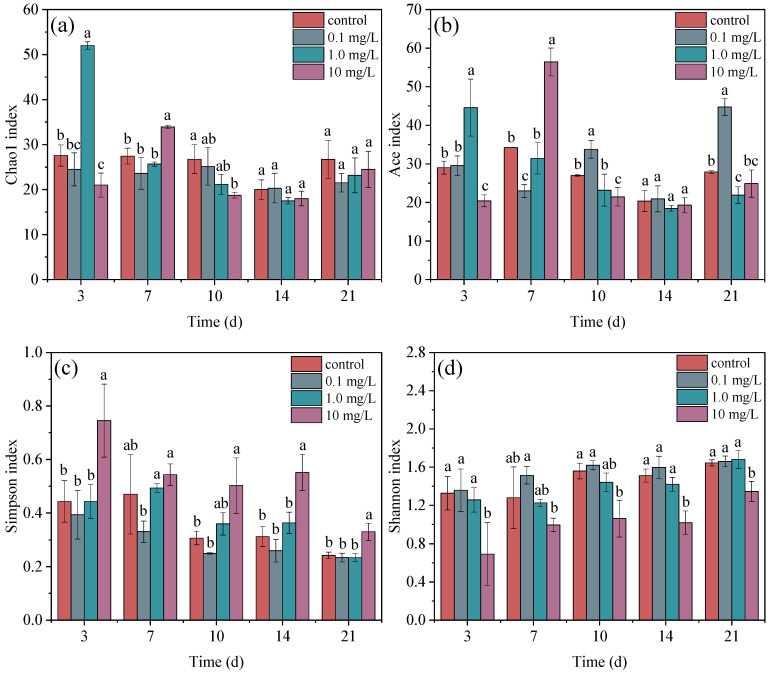
Alpha diversity ((**a**): Chao1; (**b**): Ace; (**c**): Simpson; (**d**): Shannon) of phycospheric bacteria around microalgae under ERY exposure over 21 d. Significant differences were determined according to *p* < 0.05 and are represented as different letters.

In addition to the community composition being analyzed, the alpha diversity of phycospheric bacteria was also assessed at the genus level using indices such as ACE, Chao1, Simpson, and Shannon. These indices were used to determine the richness, evenness, and diversity of the bacterial community (Figure 5). At 3 and 7 d, the Chao1 indices in the 1.0 mg/L and 10 mg/L treatment groups were significantly higher than those in the control group. However, in the later stage, there were no significant differences in Chao1 indices between the treatment groups and the control group. The Ace indices showed a similar change trend during ERY exposure over 21 d. These findings indicate that initial exposure to ERY induced an increase in the richness of phycospheric bacteria at different times; however, there were no significant differences between the treatment groups and the control group in the later exposure period. The difference in the Ace index observed in the 1.0 mg/L treatment group at 10 and 21 d may be attributed to the emergence of rare and variant species. Except for at 7 d, there were no significant differences in Simpson indices between the 0.1 mg/L treatment and the control group, or between the 1.0 mg/L treatment and the control group. During the 21 d period, the Simpson index in the 10 mg/L treatment group was closer to 1 and significantly higher than in the control group. Similarly to the Simpson index, compared to the control group, there were no significant differences in the Shannon indices for the treatments at 0.1 mg/L and 1.0 mg/L. However, the Shannon indices in the 10 mg/L treatment were significantly lower than in the control group during the 21 d. This indicates that high concentrations of ERY resulted in an uneven distribution and decreased the diversity of phycospheric bacteria. A previous study also found that antibiotics treatment slightly increased the Simpson index of the associated bacterial community of *Chrysotila roscoffensis* but without significant differences; this may be related to a lower antibiotic concentration [83]. ERY is widely used because of its antibacterial effectiveness; the occurrence of ERY is bound to affect the composition and diversity of phycospheric bacteria, especially when there are high levels of antibiotic exposure [32,84]. In contrast, antibiotic stress induced the secretion of specific metabolites in the extracellular polymeric substance of microalgae. These metabolites helped antibiotics to indirectly modulate the structure and diversity of phycospheric bacteria [81]. Therefore, in the microalgae–bacteria consortium under antibiotic exposure, the interaction between microalgae metabolism and the community composition of the bacteria determines the ecological risk of antibiotics, and even the emergence and spread of resistance genes.

### 3.6. Changes in the Metabolic and Ecological Function of Phycospheric Bacteria

**Figure 6:**
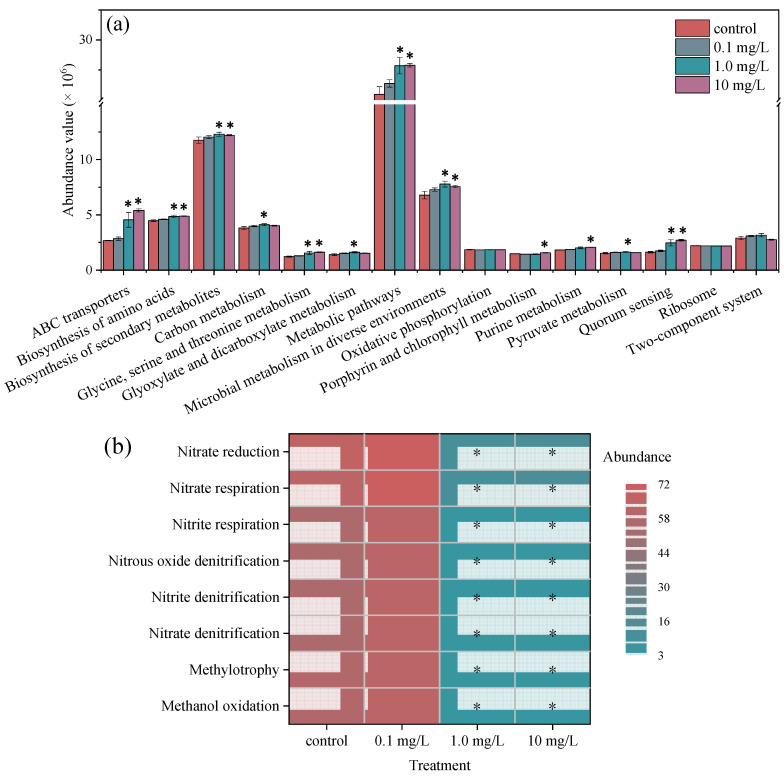
(**a**) KEGG functional pathways of phycospheric bacteria predicted by PICRUSt2. (**b**) Abundance of ecological functions of phycospheric bacteria predicted by FAPROTAX. * indicates a significant difference between the treatment and control groups (*p* < 0.05).

To better understand the effects of ERY exposure on the metabolic function of phycospheric bacteria, PICRUSt2 was used to reveal the changes in the abundance of potential functions. In Figure 6a, the top 15 functional pathways at level 3 with the highest abundance are listed. These functional pathways covered the metabolic classes of metabolism, environmental information processing, cellular processes, and genetic information processing. It is obvious that ERY increased the abundance of most metabolic pathways. The metabolic functions of ABC transporters, the biosynthesis of amino acids, the biosynthesis of secondary metabolites, glycine, serine, and threonine metabolism, metabolic pathways, microbial metabolism in diverse environments, and quorum sensing were significantly upregulated in the 1.0 and 10 mg/L treatment groups. The prediction results indicate that high concentrations of ERY altered the metabolic function of phycospheric bacteria. Previous studies have reported that ciprofloxacin exposure also increased the metabolic functions of energy metabolism, cofactor and vitamin metabolism, and the membrane transport of microbial communities [85]. Macrolides significantly upregulated the functional pathway of ribosomes, riboflavin metabolism, peptidoglycan biosynthesis, and protein export; these functions are related to amino acid metabolism and vitamin metabolism, which determine the growth and proliferation of microalgae and bacteria [86,87]. This is the result of mutual selection between microalgae and bacteria. Upregulated functions could be a useful strategy for the bacterial community against antibiotic stress, and they also provide an explanation for the observed richness without significant changes.

Meanwhile, the ecological function of phycospheric bacteria was also predicted using FAPROTAX based on the sequencing results [88]. The functions of methanol oxidation, methylotrophy, and nitrogen cycling and utilization were all inhibited in the 1.0 and 10 mg/L treatment groups. High concentrations of ERY significantly inhibited nitrate denitrification, nitrite denitrification, nitrous oxide denitrification, nitrate respiration, and nitrite respiration. The presence of high-concentration norfloxacin has been reported to impair the function of methanol oxidation, methylotrophy, and nitrate reduction in the bacterial community [89]. The predication results indicate that ERY prevented the conversion of nitrate to nitrite, ammonia, or nitrogen; this was consistent with the finding that NO_2_^−^ concentrations were reduced with increasing ERY concentrations, as shown in Figure 3b. In this study, the source of nitrogen was NaNO_3_ and Co(NO_3_)_2_·6H_2_O in the BG11 medium. The inhibited denitrification weakened the conversion of NO_3_^−^ and then ensured the absorption efficiency of nitrogen for microalgae growth in the exposure system.

## 4. Conclusions

This study investigated the growth and nitrogen metabolism of microalgae under ERY exposure, as well as analyzed and predicted the composition and function of phycospheric bacteria. Higher concentrations of ERY inhibited biomass and chlorophyll biosynthesis in the microalgae, but their growth ability gradually recovered during long-term exposure, indicating increased resistance to antibiotics. The consumption of NO_3_^−^ was consistent with microalgal growth, and the production of NO_2_^−^ was inhibited with the increase in the ERY concentration. Both microalgae and antibiotic concentrations affected the composition and diversity of phycospheric bacteria; microalgae may compete with some bacteria for resources or provide resources for them. The antibacterial properties of ERY inhibited the proliferation of sensitive bacteria. Meanwhile, phycospheric bacteria also indirectly affected microalgae growth through community function. Under ERY exposure, the bacteria exhibited enhanced metabolic functions to resist antibiotic stress and maintained a steady level of richness. High concentrations of ERY inhibited the denitrification function of phycospheric bacteria, which explains the decreased production of NO_2_^−^. The adjustment of the nitrogen metabolism of phycospheric bacteria ensured that there were sufficient nitrogen sources in the exposure system for microalgal growth. Therefore, future ecotoxicity assessments of antibiotics on microalgae should consider the effects of symbiotic microorganisms. Microalgae-based biodegradation technology should also consider the role of phycospheric bacteria, especially by nitrifying and denitrifying bacteria involved in nitrogen metabolism, which affects the microalgae’s resistance to target contaminants.

## Figures and Tables

**Table 1:** Experimental settings and the volume (mL) of each component.

Treatments	BG11 × 2	25 mg/L ERY	Sterile Water	Microalgae Strain
Control	50	0	40	10
0.1 mg/L	50	0.4	39.6	10
1.0 mg/L	50	4	36	10
10 mg/L	50	40	0	10

## Data Availability

Data will be made available on request.
